# Surgical Site Infections Caused by Highly Virulent Methicillin-Resistant *Staphylococcus aureus* Sequence Type 398, China

**DOI:** 10.3201/eid2501.171862

**Published:** 2019-01

**Authors:** Lu Sun, Yan Chen, Danying Wang, Haiping Wang, Dandan Wu, Keren Shi, Ping Yan, Yunsong Yu

**Affiliations:** Sir Run Run Shaw Hospital of Zhejiang University School of Medicine, Hangzhou, China (L. Sun, Y. Chen, D. Wang, H. Wang, D. Wu, K. Shi, Y. Yu);; Fuyang Hospital of Traumatology and Orthopedics of Traditional Chinese Medicine, Hangzhou (P. Yan);; Key Laboratory for Microbial Technology and Bioinformatics of Zhejiang Province, Hangzhou (Y. Yu)

**Keywords:** MRSA, ST398, surgical site infection, core genome MLST, virulence, bacteria, antimicrobial resistance, MRSA and other staphylococci, China, methicillin-resistant *Staphylococcus aureus*

## Abstract

We identified 2 methicillin-resistant *Staphylococcus aureus* strains of sequence type 398 from surgical site infections in China. Genetic analysis and clinical data from these strains suggested that they were human-related but sporadic. Hemolysis analysis and mouse-skin infection models indicated a high virulence potential for these strains.

Methicillin-resistant *Staphylococcus aureus* (MRSA) can cause a variety of infections, such as skin and soft tissue infection (SSTI), pneumonia, and sepsis. MRSA can also cause surgical site infections (SSI), which are the most common type of healthcare-associated infections in hospitals (*1*). In addition, MRSA infection has been reported in animals, including pigs, veal calves, and poultry (*2*). Livestock-associated MRSA (LA-MRSA) was first reported in humans in the Netherlands in 2003 (*3*), and numerous countries have subsequently reported the presence of this MRSA variant. Although LA-MRSA belongs to several clone complexes, the major clone of LA-MRSA belongs to sequence type (ST) 398 (*4*).

Several infections caused by MRSA ST398 have been reported in the community, ranging from mild skin infections to serious invasive infections, both with and without livestock contact. Fatal cases of septicemia were also reported in Japan and Denmark (*5**,**6*). MRSA ST398 isolates from human postoperative surgical site infections were presented with complete genome sequencing in Canada and in a long-term MRSA surveillance study in the United Kingdom (*7**,**8*). The virulence potential of MRSA ST398 isolated from SSI has not been reported to date.

We studied 2 MRSA ST398 strains from surgical site infections, focusing on their clinical characteristics, genetic features, and virulence potential. The study was approved by the local ethics committees in Sir Run Run Shaw Hospital with a waiver of informed consent (approval no. 20150115-1). Mouse experiments were performed with approval from the Institutional Animal Ethics Committee of Zhejiang University (approval no. ZJU2015-141-01).

## The Study

From January 2013 through December 2014, we collected a total of 147 *Staphylococcus aureus* isolates at Fuyang Hospital of Traumatology and Orthopedics of Traditional Chinese Medicine, a tertiary hospital in Hangzhou, China, with ≈500 beds. We performed multilocus sequence type (MLST) analysis on 18 SSI MRSA isolates from patients who underwent orthopedic surgery. We detected 2 MRSA strains, FY20 and FY22, which belong to ST398.

We characterized these strains by antimicrobial drug susceptibility testing, whole-genome sequencing and comparison, hemolysis analysis, and a mouse skin infection model. We included 2 community-acquired MRSA (CA-MRSA) strains isolated from patients in 2015 at Sir Run Run Shaw Hospital for comparison; SR389 was isolated from a 32-year-old female outpatient with a left neck mass, and SR411 was isolated from a 21-year-old female outpatient with lower extremity nodules and an ulcer. We sequenced the genomes on an Illumina HiSeq2000 platform (Illumina, http://www.illumina.com). We deposited the draft genome of FY20 as GenBank accession no. NTMC00000000, for FY22 as NXFU00000000, for SR389 as PDFA00000000, and for SR411 as PDFB00000000. We imported FASTA files into SeqSphere+ software version 4.1 (Ridom GmbH, http://www.ridom.de/seqsphere) for analysis (*9*). We detected virulence and resistance genes using the Center for Genomic Epidemiology website (http://www.genomicepidemiology.org).

We collected supernatants from bacterial cultures grown at 37°C for 24 h with shaking at 180 rpm. We determined hemolytic activities by incubating samples with human red blood cells (2% vol/vol in Dulbecco’s phosphate-buffered saline) for 1 h at 37°C and subsequently by measuring the optical density at 540 nm using an ELISA reader. We performed the mouse skin infection model using BALB/c female nude mice, 4–6 weeks of age. We injected anesthetized mice subcutaneously with ≈1 × 10^7^ bacterial cells in 50 μL phosphate-buffered saline in the back. We measured length and width of the abscess or lesion for 6 days with calipers, and calculated abscess size by length × width. We euthanized all mice 6 days after injection. We used South Korea CA-MRSA strain HL1 (ST72) and its mutant HL1*△agr* in the skin infection model and the hemolysis study for comparison (*10*). We performed statistical analysis using GraphPad Prism version 7.01 (GraphPad Software, https://www.graphpad.com).

We isolated both MRSA strains from SSIs, FY20 and FY22. FY20 was isolated from a 42-year-old female patient who had undergone steel plate–screw internal fixation after a right tibial plateau comminuted fracture in May 2014. She was discharged in June 2014 and admitted to the hospital again because of swelling and pain in her right knee 71 days after surgery. The abscess was surgically debrided, and she was treated with levofloxacin. We isolated FY22 from a 52-year-old male patient. This patient also underwent steel plate–screw internal fixation after a right tibial plateau comminuted fracture in August 2014. He was discharged in September 2014 and was admitted to the hospital again for pus and exudate from the incision site in the right knee 21 days after surgery. The wound was surgically debrided, and he was treated with cefuroxime and levofloxacin. These 2 patients were admitted to different wards and were treated by different teams during hospitalization. 

**Figure 1:**
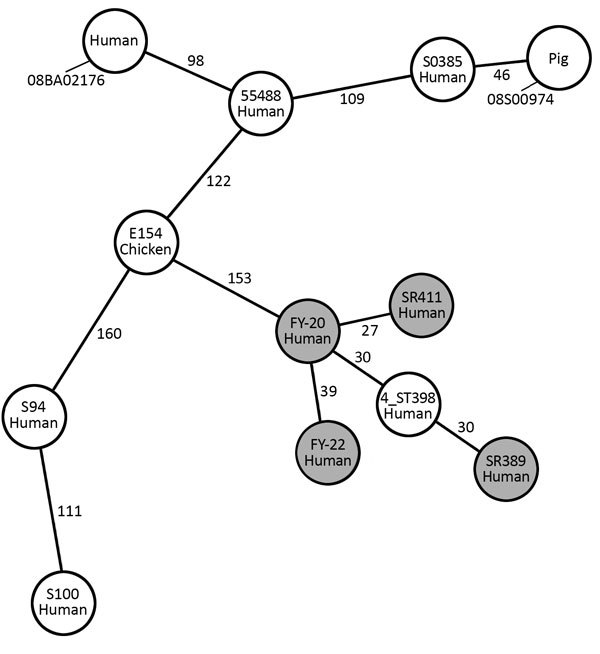
Minimum-spanning tree built from the core genome multilocus sequence type allelic profiles of MRSA ST398 strains from 4 patients in China (gray circles) and other ST398 strains. Each circle represents a single strain and is named with the sample and the origin. The 12 strains are based on 1,807 columns with the pairwise ignoring missing values option in Ridom SeqSphere+ software (Ridom GmbH, http://www.ridom.de/seqsphere). The numbers on the connecting lines indicate the number of allelic differences between 2 strains. *S. aureus* strain COL (GenBank accession no. NC_002951) is used as a reference. S0385 (human, MRSA, NC_017333.1), 08BA02176 (human, MRSA, CP003808.1), 55488 (human, MRSA, NZ_LAWV00000000), 4_ST398 (human, MRSA, He L et al, 2018), S94 (human, MSSA, AUPW00000000), S100 (human, MSSA, AUPV00000000), 08S00974 (animal, MRSA, NZ_CP020019.1), and E154 (animal, MRSA, CP013218.1) are used for comparison. MRSA, methicillin-resistant *Staphylococcus aureus*; ST, sequence type.

Antimicrobial susceptibility testing results showed that the 4 ST398 strains in our study had a similar resistance phenotype; they displayed resistance to β-lactam antimicrobial drugs but were susceptible to most other antimicrobial drugs. FY20 and SR411 were resistant to clindamycin and erythromycin. Resistance genes detected using the Center for Genomic Epidemiology site matched the resistance phenotypes (Table). The 4 ST398 strains were negative for *tetM* and *czrC*, were positive for φSa3, and displayed susceptibility to tetracycline. We classified FY20, FY22, and SR389 into SCC*mec* V and *spa*-type t034, and SR411 into SCC*mec* V and *spa*-type t1255. We built a minimum-spanning tree based on the core genome MLST (cgMLST) allelic profiles (Figure 1). There were 27 allelic differences by cgMLST analysis between FY20 and SR411; for 5 pairs of other strains from our study (FY20 and FY22, FY20 and SR389, FY22 and SR389, FY22 and SR411, and SR389 and SR411), there were >30 allelic differences. From a previous study, 9–29 allelic differences can be considered possibly related, whereas *>*30 are considered unrelated (*11*). When we compared the strains in our study to MRSA ST398 strain 4_ST398 from a hospital in Shanghai (*12*), we found 30–40 allelic differences between each of our 4 strains and the reference strain. There were >150 allelic differences between the SSI strains in our study and other ST398 strains, including animal- and human-related strains, from other studies (*7**,**13**,**14*).

**Table:** Characteristics of methicillin-resistant *Staphylococcus aureus* ST398 isolates from 4 patients, China*

Characteristic	Isolate			
	FY20	FY22	SR389	SR411
Patient age, y/sex	42/F	52/M	32/F	21/F
Admission diagnosis	Right tibial plateau comminuted fracture	Right tibial plateau comminuted fracture	Left neck mass	Lower extremity nodules with ulceration
Infection type	SSI	SSI	SSTI	SSTI
SCC*mec *type	V	V	V	V
*spa* type	t034	t034	t034	t1255
Panton–Valentine leukocidin	–	–	–	–
Resistance phenotype	PEN, OXA, CLI, ERY	PEN, OXA	PEN, OXA	PEN, OXA, CLI, ERY
Resistance genes	*mecA*, *blaZ*, *ermC*	*mecA*, *blaZ*	*mecA*, *blaZ*	*mecA*, *blaZ*, *ermC*
Virulence factors	aur, sak, scn, hlb, hlgA, hlgB, hlgC	aur, sak, scn, hlb, hlgA, hlgB, hlgC	aur, sak, scn, hlb, hlgA, hlgB, hlgC	aur, sak, scn, hlb, hlgA, hlgB, hlgC

*aur, aureolysin; CLI, clindamycin; ERY, erythromycin; hlb, β-hemolysin; hlgA, gamma-hemolysin component; OXA, oxacillin; PEN, penicillin; sak, staphylokinase; scn, staphylococcal complement inhibitor; SCC, staphylococcal cassette chromosome; SSI, surgical site infection; SSTI, skin and soft tissue infection; –, negative.

**Figure 2:**
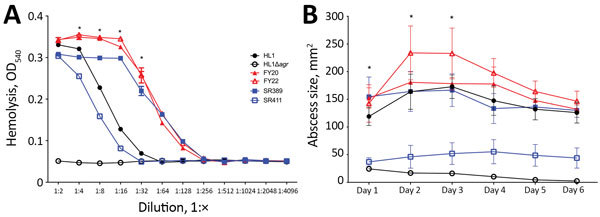
Virulence phenotype of MRSA ST398 isolates. A) Hemolysis analysis. The hemolytic activity of culture filtrates grown for 24 h was measured in triplicate at given dilutions. The mean and SE are shown. The statistical analysis used a 2-way analysis of variance between multiple groups. FY20 and FY22 were significantly stronger than other strains in dilution 1:4, 1:8, 1:16, and 1:32 (p<0.05). B) Abscess sizes in the mouse skin infection model. There were 4 mice per strain, and the mean and SE are shown. The statistical analysis used a 2-way analysis of variance to compare data for multiple groups. The abscess sizes of FY20 and FY22 were significantly larger than SR411 and the HL1 *agr* mutant (p<0.05) but were similar to SR389 and HL1 (p>0.05) at day 1, day 2, and day 3. MRSA, methicillin-resistant *Staphylococcus aureus*; OD, optical density; ST, sequence type.

All 4 strains in our study contained the same virulence factors (Table). We analyzed the lysis of human erythrocytes, which is a key determinant of *S. aureus* virulence. Erythrocyte lysis by SR389, FY20, and FY22 was stronger than for CA-MRSA HL1 (p<0.05) (Figure 2, panel A), whereas SR411 showed a relatively weaker lytic capacity. We used a mouse skin infection model to evaluate the virulence potential of ST398 strains in vivo. In this model, abscesses caused by SSI strains (FY20 and FY22) were significantly larger than those caused by SR411 and the HL1 *agr* mutant (p<0.05) and were similar to those caused by SR389 and HL1 (p>0.05) (Figure 2, panel B). These findings demonstrated the pronounced virulence potential of SSI ST398 strains to cause invasive skin infections similar to those associated with HL1, which was a predominant CA-MRSA clone in South Korea.

## Conclusions

We reported 2 ST398 MRSA strains causing SSI, which were classified into hospital-acquired MRSA according to the Centers for Disease Control and Prevention definition (*15*). The clinical data and cgMLST results indicated no clonal transmission in these 2 cases. These 2 patients denied livestock contact, and further genetic analysis showed characteristics of human-associated isolates. It is possible that the 2 patients were colonized with ST398 MRSA in the community and their infections developed later, after surgery. A limitation of this study is that we detected only 2 ST398 HA-MRSA strains, which means that it is difficult to evaluate the spreading trend and the threat of this lineage in the healthcare setting. However, our results revealed the emergence and transmission pattern of ST398 MRSA in the surgical department of a hospital in China.
